# Single-cell transcriptomic analysis of neuroepithelial cells and other cell types of the gills of zebrafish (*Danio rerio*) exposed to hypoxia

**DOI:** 10.1038/s41598-022-13693-1

**Published:** 2022-06-16

**Authors:** Wen Pan, Rafael Soares Godoy, David P. Cook, Angela L. Scott, Colin A. Nurse, Michael G. Jonz

**Affiliations:** 1 Department of Biology, University of Ottawa, 30 Marie Curie Pvt., Ottawa, ON K1N 6N5 Canada; 2 Sinclair Centre for Regenerative Medicine, Ottawa Hospital Research Institute, Ottawa, ON K1H 8L6 Canada; 3 Cancer Therapeutics Program, Ottawa Hospital Research Institute, Ottawa, ON K1H 8L6 Canada; 4 Department of Cellular and Molecular Medicine, University of Ottawa, Ottawa, ON K1H 8M5 Canada; 5 Department of Pathology and Molecular Medicine, McMaster University, Hamilton, ON L8S 4L8 Canada; 6 Department of Biology, McMaster University, Hamilton, ON L8S 4K1 Canada; 7 Brain and Mind Research Institute, University of Ottawa, Ottawa, ON K1H 8M5 Canada

**Keywords:** Zebrafish, RNA sequencing, Peripheral nervous system, Sensory processing, Cell biology, Molecular biology, Physiology, Respiration, Zoology, Animal physiology

## Abstract

The fish gill is a multifunctional organ involved in numerous physiological processes, such as gas exchange and sensing of hypoxia by respiratory chemoreceptors, called neuroepithelial cells (NECs). Many studies have focused on zebrafish (*Danio rerio*) to investigate the structure, function and development of the gills, yet the transcriptomic profile of most gill cells remains obscure. We present the results of a comprehensive transcriptomic analysis of the gills of zebrafish using single-cell RNA sequencing (scRNA‐seq). Gill cells from *ETvmat2:EGFP* zebrafish were individually labelled before scRNA‐seq library construction using 10× Genomics Chromium technology. 12,819 cells were sequenced with an average depth of over 27,000 reads per cell. We identified a median of 485 genes per cell and 16 cell clusters, including NECs, neurons, pavement cells, endothelial cells and mitochondrion-rich cells. The identity of NECs was confirmed by expression of *slc18a2*, encoding the vesicular monoamine transporter, Vmat2. Highly differentially-expressed genes in NECs included *tph1a*, encoding tryptophan hydroxylase, *sv2* (synaptic vesicle protein), and proteins implicated in O_2_ sensing (*ndufa4l2a*, *cox8al* and *epas1a*). In addition, NECs and neurons expressed genes encoding transmembrane receptors for serotonergic, cholinergic or dopaminergic neurotransmission. Differential expression analysis showed a clear shift in the transcriptome of NECs following 14 days of acclimation to hypoxia. NECs in the hypoxia group showed high expression of genes involved in cell cycle control and proliferation. The present article provides a complete cell atlas for the zebrafish gill and serves as a platform for future studies investigating the molecular biology and physiology of this organ.

## Introduction

The fish gill is a complex, multifunctional organ that orchestrates numerous physiological processes, including respiratory gas exchange, ionic and acid–base regulation, and excretion of nitrogenous compounds^1^. The gills are organized into four branchial arches (in teleosts) with numerous filaments, each of which contains afferent and efferent arteries and gives rise to a series of respiratory lamellae. The gills are covered by an epithelial layer comprised of a multitude of cell types. Those that are well documented include pavement (respiratory) cells, chemoreceptors, neurons, mitochondrion-rich cells (ionocytes) and pillar cells^1,2^. Despite decades of studies on the gills, including those of the model vertebrate, zebrafish (*Danio rerio*), the regulation of gene expression in gill cells remains largely unknown. We present the first transcriptomic analysis of cells from the zebrafish gills using single-cell RNA sequencing (scRNA-seq). We identified hundreds of genes within each cell and defined 16 separate cell types based on differential gene expression. The present study provides a complete atlas of gill cells from zebrafish with a particular focus on cells associated with O_2_ sensing and the control of respiration.

As part of its role as a respiratory organ, the gill filaments are the primary site for respiratory chemoreceptors—called neuroepithelial cells or neuroendocrine cells (NECs)—due to their resemblance to neurons, their presence within the epithelial layer, and their secretory activity. NECs detect changes in O_2_ and CO_2_/H^+^ and display K^+^ channel inhibition, membrane depolarization and synaptic vesicle recycling under conditions of hypoxia or hypercapnia/acidosis^3–8^. These cellular responses are believed to initiate reflex physiological changes, such as hyperventilation, thus maintaining internal O_2_ homeostasis^9,10^. NECs contain the neurotransmitter, serotonin (5-hydroxytryptamine, 5-HT), stored in synaptic vesicles and are innervated by sensory nerve fibres^11,12^.

The first gill arch and associated NECs display evidence of homology with the carotid body, the primary O_2_ sensing organ in mammals^9^, though recent evidence suggests that NECs may be homologues of pulmonary neuroepithelial bodies, which function as respiratory chemoreceptors during the perinatal period^13,14^. Like mammalian chemoreceptors, NECs display pronounced plasticity when acclimated to hypoxia, such as hypertrophy^3,6,15^, proliferation and changes in gene expression^16^. In addition to 5-HT, other neurotransmitters, such as acetylcholine (ACh), adenosine triphosphate (ATP) and dopamine (DA), appear to be involved in hypoxic signalling in the gill^17–21^; however, the location or expression of corresponding receptor targets have not been identified.

Despite recent advances in delineating the role of gill NECs in the hypoxic response, specific mechanisms through which NECs sense, transduce and transmit hypoxic signals remain ambiguous. Conventional molecular approaches, such as polymerase chain reaction (PCR) analysis, allow only few gene candidates to be examined at a time, making it difficult to draw connections between different components of the signal pathway. With respect to the carotid body, microarray analysis and/or real-time quantitative PCR was used to characterize genes involved in the O_2_-sensing pathway in both mouse^22^ and human^23^, whereas scRNA-seq analysis revealed the first transcriptomic profile of mouse chemoreceptor cells, highlighting genes likely to be important in chemosensory function^24^. A comprehensive transcriptomic analysis, such as RNA sequencing, provides more specificity in gene detection, identifies more protein-coding genes, and provides a wider range of gene expression changes when compared to microarrays^25^. In addition, NECs make up only a small proportion of gill cells—about 0.79–1.17% of all dissociated cells from distal filaments in zebrafish, depending on whether animals are exposed to normal levels of O_2_ or hypoxia^16^. Consequently, the whole-tissue approach to transcriptomic analysis would limit collection of sufficient amounts of RNA from the relatively small population of gill NECs.

In the present study, we employed the droplet-based scRNA-seq approach to profile individual cells dissociated from the gills of zebrafish under control conditions or acclimated to 2 weeks of hypoxia. This approach was adopted to capture transcriptomes of individual NECs, sensory neurons involved in the hypoxia signalling pathway, and other cell types of the gills. Cell types were identified based on marker gene expression, and a gill cell atlas was generated from our dataset. Cross examination of different cell clusters uncovered genes that were highly differentially expressed in NECs, many of which are shared by chemoreceptor (type I) cells of the mammalian carotid body. Our data provide evidence that NECs exhibit the machinery for chemosensory transduction of hypoxic signals. Our results also reveal the expression of 5-HT, ACh and DA receptors in distinct cell types in the gills, indicating their potential roles in O_2_-chemosensory transmission. Lastly, 2 weeks of exposure to hypoxia affected the population size and transcriptome of gill cells, including NECs. NECs had increased expression of genes involved in protein synthesis, cell growth and control of the cell cycle.

## Results

### Construction of the gill cell atlas

**Figure 1:**
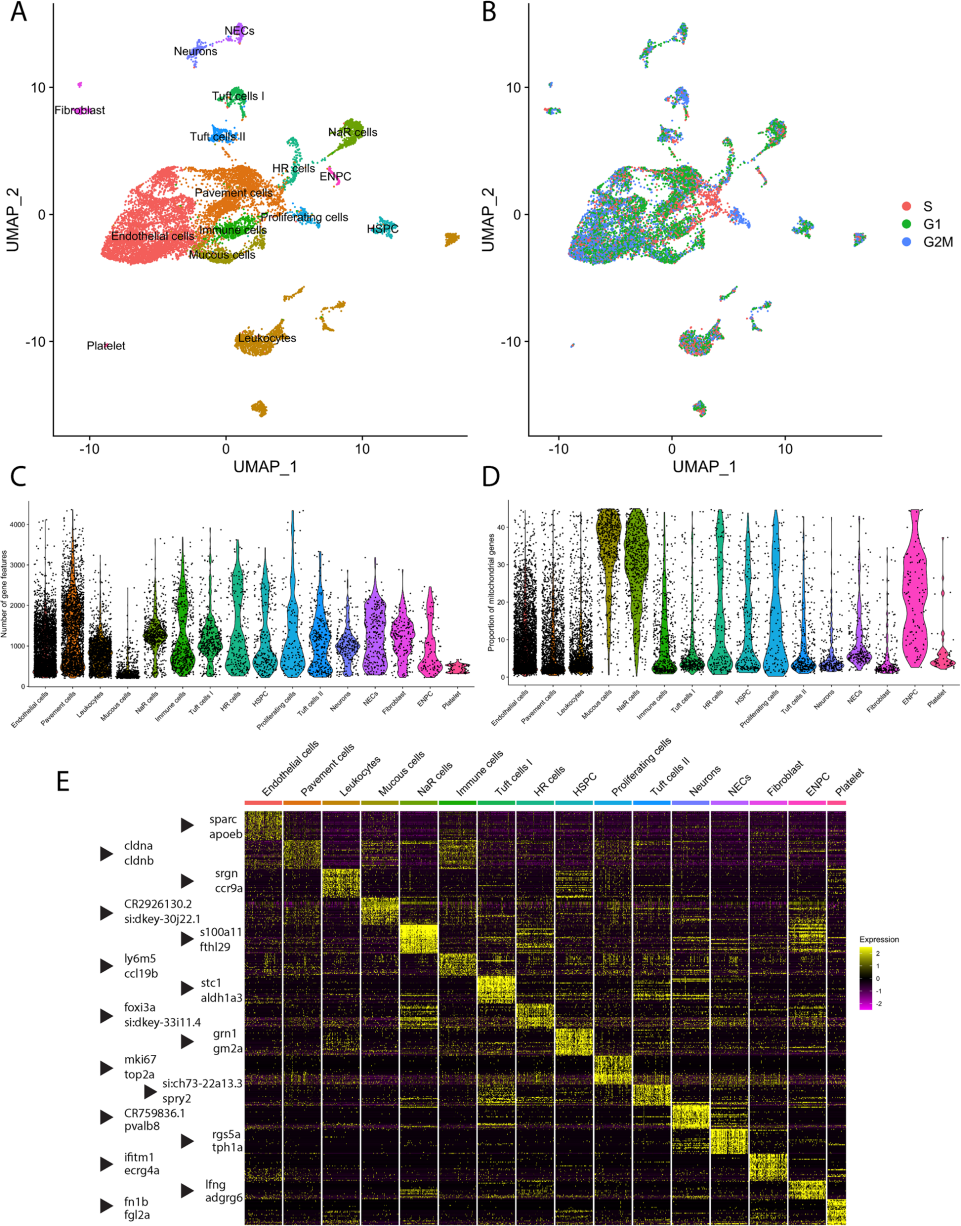
Transcriptomic atlas of gill cells dissociated from the gills of *ETvmat2:EGFP* zebrafish. (**A**) Unsupervised uniform manifold approximation projection (UMAP) plot in which gill cells were subdivided into 16 clusters. Colours correspond to separate cell clusters whose identities were defined by the major cell type present within the cluster. Identified cell clusters included (organized by decreasing abundance) endothelial cells, pavement cells, leukocytes, mucous cells, Na^+^/K^+^-ATPase-rich cells (NaR cells), immune cells, tuft cells I, H^+^-ATPase-rich cells (HR cells), hematopoietic stem and progenitor cells (HSPC), proliferating cells, tuft cells II, neurons, neuroepithelial cells (NECs), fibroblasts, epidermal neural progenitor cells (ENPC) and platelets. (**B**) UMAP visualization of cell-cycle phases of the aforementioned clusters. G1, G1 phase; G2M, G2 and metaphase; S, synthesis phase. (**C**) Violin plot showing the number of unique genes in each cell cluster. (**D**) Violin plot showing the proportion of mitochondrial genes present within each cell cluster. (**E**) Heatmap of the top 32 genes that were differentially enriched in each cell cluster. Two representative genes from each cluster are indicated by an arrowhead. Scaled gene expression is shown by colours, where yellow indicates relatively high expression and purple indicates relatively low expression.

Gill cells were dissociated from a total of 8 zebrafish: 4 fish exposed to hypoxia and 4 fish in normoxia as controls. After quality filters and doublet removal, a total of 12,819 cells were contained in the dataset. We then generated a gill cell atlas with 16 cell identities using our single-cell sequencing data (Fig. 1A,B). The data had a mean of 27,312 reads per cell, a median of 485 genes per cell and a median of 2844 unique transcript molecules per cell. Following demultiplexing of sample barcodes, the multidimensional dataset was mapped onto a two-dimensional graph using the Uniform Manifold Approximation Projection (UMAP) method. This atlas served as a tool to demonstrate relationships between cell clusters and visualize relative gene expression.

The cell atlas was built with integrated data from both treatment conditions to ensure consistent clustering and cell identification between conditions. Unsupervised clustering analysis compared cells based on transcriptomic similarities and returned 22 clusters. The resulting cell atlas contained 16 cell identities, including NECs, neurons and other cell types, as shown in Fig. 1A (see also Supplementary Table S1). Based on our knowledge of gill cell morphology and gene expression, certain cell clusters were annotated with a broad identity without further specification. For example, two computationally-defined cell clusters expressing endothelial cell markers could not be further specified based on gene expression. These clusters were therefore combined into a single cluster. Annotated cell populations had a variable number of unique genes and mitochondrial gene content (Fig. 1C,D). Moreover, the 16 cell identities differed greatly in gene expression. Their highly differentially expressed genes are depicted in the heatmap in Fig. 1E.

The annotation of cell clusters was mostly based on previously established molecular markers and known functional characteristics of gill cells. For example, NECs displayed particularly high expression for *tph1a* (Fig. 1E), the gene encoding the rate-limiting enzyme in the synthesis of serotonin. NECs are the major source of serotonin in the zebrafish gill^12^. Endothelial cells were identified based on their high expression of endothelial PAS domain protein (*epas1a* and *epas1b*), basal cell adhesion molecule (*bcam*) and endothelin 1 (*edn1*)^26,27^. The endothelial cell cluster may include pillar cells that form the vascular channels of the respiratory lamellae. Pillar cells are a modified form of endothelial cells^2^. The gene, *ptprc*, encoding the protein, tyrosine phosphatase receptor type C or CD45 antigen, was used to identify the broad leukocyte population^28^. The cluster of mucous cells did not have high expression of specific cell markers but was identified based on expression of mucins (*muc5.1* and *muc13a*).

For clusters without established gene markers, the top 32 significantly enriched genes were examined in more detail to reveal their identity. For instance, respiratory pavement cells were identified by their high expression of *cldna, cldnb,* and *cldne* (Fig. 1E), genes encoding claudin proteins important for establishing tight junctions. These genes are orthologous to the human gene, *CLDN4*, which is highly expressed in respiratory type I and type II alveolar cells in lung epithelium^29^. Immune cells were assigned this identity due to their high expression of lymphocyte antigen *ly6m5* (Fig. 1E) and chemokines, such as *ccl19b* (Fig. 1E), *cxcl8a* and *ccl25b.* In addition, proliferating cells were identified based on their high expression of proliferative markers, such as *mki67* and *top2a* (Fig. 1E)*.* Results from cell cycle scoring confirmed that cells in the proliferating cell cluster were in growth phase 2 or metaphase (G2M) of the cell cycle (Fig. 1B). Moreover, two stem and progenitor cell populations were identified in our data. They are the cluster of hematopoietic stem and progenitor cells (HSPC), identified by their expression of *grn1* (Fig. 1E), *grn2* and *thy1/*cd90^30,31^, and the cluster of epidermal neural progenitor cells (ENPC), which form ionocytes in zebrafish embryos, identified by the expression of *lfng* (Fig. 1E)*, notch 1b, grhl1* and *foxi3b*^32,33^*.*

Two populations of ionocytes, cells involved in ion transport in the gills, were identified. H^+^-ATPase-rich cells (HR cells) and Na^+^/K^+^-ATPase-rich cells (NaR cells) were identified in our dataset through their expression of *foxi3a* and *foxi3b* (Fig. 1E). These genes were present in both computationally-defined clusters. Close examination of the top differentially expressed genes in these two cell clusters showed that one expressed a high level of *atp6v1c1b* and *atp6v1aa* encoding H^+^-ATPase subunits. These cells were further identified as HR cells^34^. The other cluster expressed a high level of *atp1b1b* and *atp1a1a.1* for Na^+^/K^+^ ATPase subunits, markers for NaR cells^35^ and were annotated as such. Moreover, NaR cells showed high mitochondrial gene content, as shown in Fig. 1D, and this feature is consistent with the high mitochondrial content of mitochondrion-rich ionocytes^1^.

Furthermore, we have identified novel cell populations in the gills. Two cell clusters expressing significant levels of the transcription factor, *pou2f3*, advillin (*avil*) and villin 1 (*vil1*) were annotated with identities, tuft cells I and tuft cells II. These cells were given such identities due to their resemblance in gene expression to mammalian tuft cells, which display a tuft-like brush of microvilli^36^. Tuft cells found in the respiratory tract, in close proximity to pulmonary neuroendocrine cells in lungs, represent an epithelial cell type with both sensory and secretory properties^37^. Genes orthologous to mammalian tuft cell markers, such as *mydgf, ptgs2b,* and *gng13a* were highly expressed in tuft cells I, whereas *mydgf*, *ptgs2b*, *trpm4a* and *alox5a* were highly expressed in tuft cells II. Both clusters shared distinctly high expression of *plcg2*.

### Identification of O_2_-chemoreceptive neuroepithelial cells and their expression of unique markers

**Figure 2:**
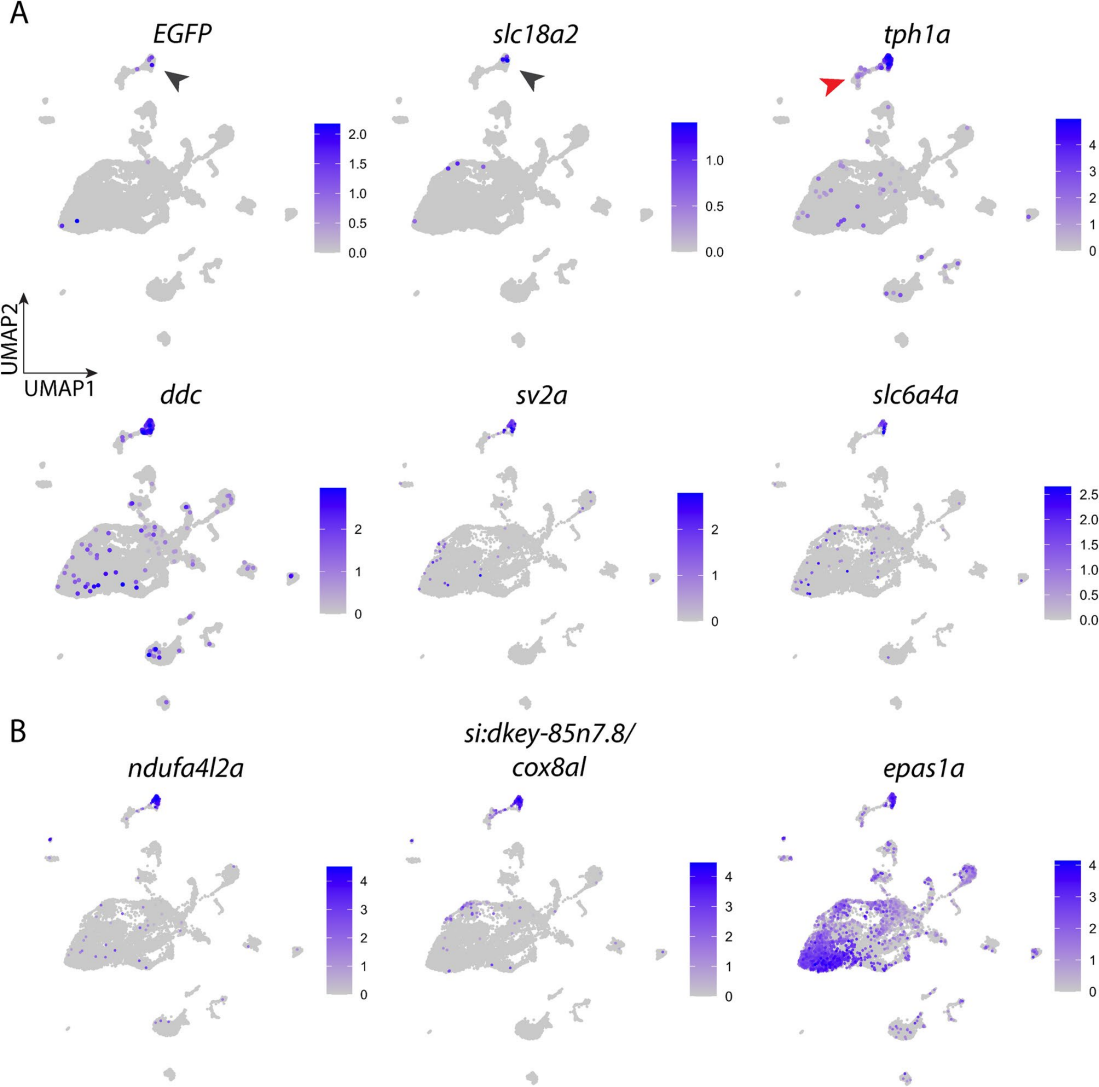
Expression of signature genes found in neuroepithelial cells (NECs). (**A**) UMAP plots showing the log-normalized counts of selective genes involved in identification of NECs. Colour intensity is proportional to the expression level of a given gene. Scales indicate relative gene expression. Black arrowheads indicate high expression in the NEC cluster; red arrowhead indicates expression in the neuron cluster. (**B**) UMAP plots showing the normalized counts of representative genes that may be involved in O_2_ sensing in NECs. See text and Table 1 for gene description.

Neuroepithelial cells of the gills were identified based on expression of the reporter genes, *EGFP* and *slc18a2*, the latter of which encodes the vesicular monoamine transporter, Vmat2 (black arrowheads, Fig. 2A). We previously established that *EGFP* recapitulates the expression of *slc18a2* in the *ETvmat2:EGFP* transgenic line, and expression of both genes in the gills was limited only to serotonergic NECs^16^. Moreover, high expression of *tph1a* and DOPA decarboxylase (*ddc*) indicates a serotonergic phenotype in these cells*. tph1a* and *ddc* were expressed predominantly in the NEC cluster (Fig. 2A) with an average log-fold increase of 3.42 and 1.29, respectively, in NECs compared with other gill cells. In addition, some cells in the neuron cluster adjacent to NECs in the UMAP plot also expressed low levels of *tph1a* (red arrowhead, Fig. 2A), consistent with serotonergic neurons of the gill filaments^12^. Moreover, the synaptic vesicle glycoprotein 2A (*sv2a*) and the serotonin transporter, SERT (*slc6a4a*), were highly differentially expressed in NECs (Fig. 2A), providing additional verification.

In the cell atlas, NECs were separated from other cell clusters, illustrating their unique transcriptomic profile. Differential gene expression (DE) analysis of NECs against all other gill cells in the dataset indicated that 617 genes were differentially expressed in NECs (based on adjusted P-value < 0.05). The most differentially expressed genes in NECs are listed in Table 1. Among these, regulators of G-protein signaling, such as *si:ch211-196h16.12* (encoding regulator of G-protein signaling 5-like, Rgs5al)*, rgs5a* and *rgs4*, were among the most differentially expressed and were up to a 6 log-fold increase from the global average. A few G-protein genes, such as *si:dkey-27j5.5* and *rasd1*, were also highly expressed in NECs. NECs also had exclusively high expression of gastrin releasing peptide, *grp*, and retinaldehyde dehydrogenase 2, *aldh1a2*, for neuropeptide signaling and retinoic acid production, respectively. In addition, a few transcription factors, *zgc:56628*, *pbx1a*, *meis2a.1*, *ascl1a* and *tbx1*, were uniquely expressed in NECs. Moreover, the glial cell line-derived neurotrophic factor receptor, *gfra3*, was found to be highly expressed. NECs also expressed genes of the *kcn* family of voltage-gated K^+^ channels, including *kcnd2* and *kcnip3b*, the latter of which encodes the channel-interacting protein, calsenilin (Supplementary Table S1).

**Table 1:** The top 25 most abundant genes in the neuroepithelial cell (NEC) cluster.

Rank	Gene	Gene name or encoded protein	Avg. log-fold change
1	*si:ch211-196h16.12*	Regulator of G protein signaling 5-like	6.67
2	*rgs5a*	Regulator of G protein signaling 5a	4.23
3	*grp*	Gastrin-releasing peptide	3.48
4	*tph1a*	Tryptophan hydroxylase 1 (tryptophan 5-monooxygenase) a	3.42
5	*ndufa4l2a*	NADH dehydrogenase [ubiquinone] 1α subcomplex subunit 4-like 2a	3.34
6	*aldh1a2*	Retinal Dehydrogenase 2	2.82
7	*si:dkey-85n7.8*	Cytochrome c oxidase subunit 8A, mitochondrial-like	2.79
8	*si:dkey-27j5.5*	GTP-binding protein Rhes	2.63
9	*scgn*	Secretagogin, EF-hand calcium binding protein	2.62
10	*CABZ01030107.1*	CABZ01030107.1 (Clone-based (Ensembl) gene)	2.41
11	*atp1a3b*	ATPase Na^+^/K^+^ transporting subunit alpha 3b	2.37
12	*col18a1a*	Collagen type XVIII alpha 1 chain a	2.36
13	*atp1b2a*	ATPase Na^+^/K^+^ transporting subunit beta 2a	2.26
14	*rgs4*	Regulator of G protein signaling 4	2.16
15	*zgc:56628*	LIM domain transcription factor LMO4	2.16
16	*vamp2*	Vesicle-associated membrane protein 2	2.02
17	*syt1a*	Synaptotagmin Ia	1.97
18	*pbx1a*	Pre-B-cell leukemia homeobox 1a	1.94
19	*rasd1*	RAS, dexamethasone-induced 1	1.93
20	*scg3*	Secretogranin III	1.86
21	*gfra3*	GDNF family receptor alpha 3	1.83
22	*cplx2*	Complexin 2	1.82
23	*tnnc1b*	Troponin C type 1b (slow)	1.81
24	*meis2a.1*	Meis homeobox 2a	1.78
25	*qdpra*	Quinoid dihydropteridine reductase a	1.76

Average log-fold change indicates higher expression over other cell types. The adjusted P-value for each gene was < 0.05. See Supplementary Table S1 for the full list of genes within each cell cluster.

Gill NECs are known for their O_2_ sensing properties^3–8^. To investigate potential molecular O_2_ sensors within zebrafish NECs, we examined top expressing mitochondrial genes in the NEC cluster since a signature profile of mitochondrial gene expression has been linked to the exquisite O_2_ sensitivity of carotid body chemoreceptor type I cells^22^. We found the NADH dehydrogenase (ubiquinone) subunit (*ndufa4l2a*) and the cytochrome c oxidase subunit 8A-like (*si:dkey-85n7.8*) to be highly expressed in NECs (Fig. 2B; Table 1). *ndufa4l2a* was ranked as the fifth differentially expressed gene with an average log-fold increase of 3.34, and *si:dkey-85n7.8* was ranked seventh with an average log-fold increase of 2.79 in NECs. As demonstrated in the cell atlas, these high expression values were unique to NECs, as their expression in other cell clusters was much lower (Fig. 2B). Moreover, NECs also showed high expression of hypoxia inducible factor genes, *epas1a*/*hif-2aa* and *epas1b/hif-2a*. In particular, *epas1a*/*hif-2aa* was highly differentially expressed in NEC and endothelial cell clusters, and was found at lower levels of expression in other cell types (Fig. 2B). The genes, *rgs4* and *rgs5a*, encode regulator of G-protein signaling proteins and were highly expressed in NECs (see Fig. 1E and Table 1). Both *Rgs4* and *Rgs5* were shown to be targets of HIF in carotid body type I cells^24^, and *Rgs5a* a target of Hif2α in O_2_-sensitive adrenal medullary chromaffin cells^38^. Thus, high expression of *rgs4* and *rgs5a* in zebrafish NECs may be related to high endogenous expression of *hif2α*.

NECs also expressed *slc16a3* at a significantly higher level than other cell types, though at a lower log-fold change of 0.53 (Supplementary Table S1). This gene encodes the monocarboxylate transporter MCT4, which has recently been implicated in a lactate-sensing mechanism in carotid body type I cells^39^, and there is evidence that gill NECs participate in lactate sensing in rainbow trout (*Oncorhynchus mykiss*)^40^.

### Identification of the gill neuron cluster

**Figure 3:**
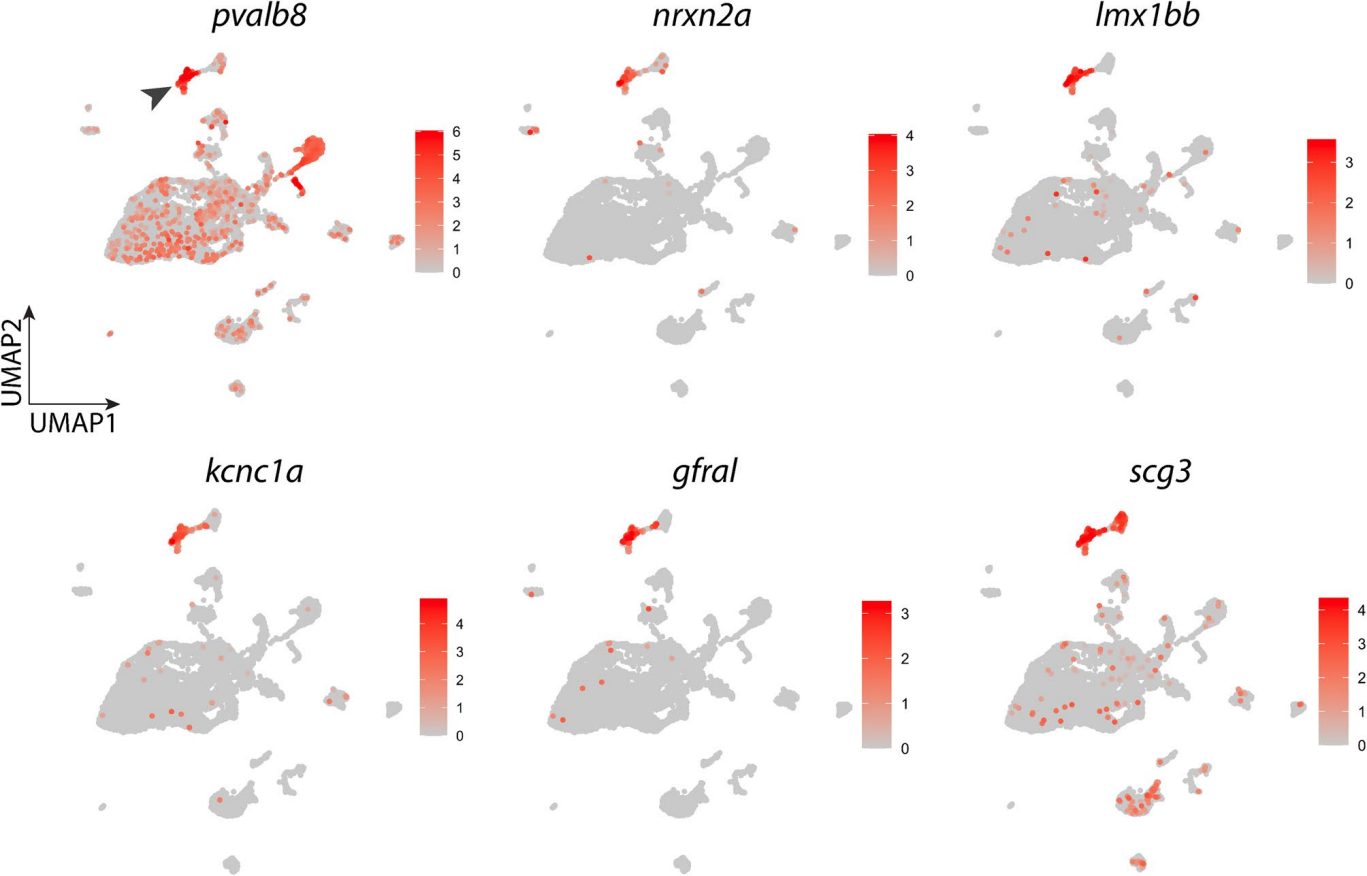
Expression of signature genes found in the neuron cluster. UMAP plots showing the log-normalized counts of selective genes that were highly differentially expressed in neurons. Colour intensity is proportional to the expression level of a given gene. Scales indicate relative gene expression. Arrowhead indicates significant expression in the neuron cluster. See text and Table 2 for gene description.

The neuron cluster in the cell atlas expressed high levels of neuronal markers, such as *pvalb8* and *nrxn2a* (arrowhead, Fig. 3). *pvalb8* encodes parvalbumin 8 and is highly expressed in GABAergic interneurons in the rodent brain^41^. However, we did not detect significant expression of genes involved in gamma-aminobutyric acid (GABA) synthesis or uptake in the neuron cluster. Neurons also showed high expression of transcription factors, *lmx1bb* and *lmx1ba*, and their mammalian orthologues were shown to be important to survival of dopaminergic neurons in mammals^42^. In addition, voltage-gated K^+^ channels (*kcnc1a*), GDNF family receptor alpha-like (*gfral*) and secretogranin III (*scg3*) were among the top differentially expressed genes in neurons (Fig. 3). GFRAL and SCG3 were shown to be widely expressed in neuronal cells^43,44^. Differential expression (DE) analysis of neurons against all other gill cells showed 501 genes, whose expression was significantly higher in neurons (adjusted P-value < 0.05). The top differentially expressed genes in neurons are listed in Table 2. Other genes that were highly expressed in neurons include *prr15la, vill* and *stom.* Neurons also uniquely expressed transcription factors, *foxa, foxa1* and *foxa3.* In addition, genes encoding Ca^2+^-binding proteins, *ano2b* and *anxa5b*, were highly differentially expressed in neurons.

**Table 2:** The top 25 most abundant genes in the neuron cluster.

Rank	Gene	Gene name or encoded protein	Avg. log-fold change
1	*CR759836.1*	5-Hydroxytryptamine receptor 3A-like	4.06
2	*pvalb8*	Parvalbumin 8	3.74
3	*si:ch211-152c2.3*	si:ch211-152c2.3	3.14
4	*scg3*	Secretogranin III	2.98
5	*kcnc1a*	Potassium voltage-gated channel, Shaw-related subfamily, member 1a	2.56
6	*prr15la*	Proline rich 15 like a	2.29
7	*lmx1bb*	LIM homeobox transcription factor 1, beta b	2.24
8	*vill*	Villin-like	2.13
9	*stom*	Stomatin	1.99
10	*ano2b*	Anoctamin 2b	1.96
11	*si:ch211-207j7.2*	Protein IWS1 homolog A-like	1.90
12	*syt1a*	SYNAPTOTAGMIN Ia	1.89
13	*zgc:112408*	zgc:112408	1.85
14	*hepacam2*	HEPACAM family member 2	1.79
15	*gfral*	GDNF family receptor alpha like	1.77
16	*nrxn2a*	Neurexin 2a	1.66
17	*rbfox1*	RNA binding fox-1 homolog 1	1.65
18	*zgc:101731*	SNARE_SNAP25N and SNARE_SNAP23C domain-containing protein; synaptosomal-associated protein 25	1.62
19	*anks4b*	Ankyrin repeat and sterile alpha motif domain containing 4B	1.61
20	*itpkca*	Inositol-trisphosphate 3-kinase Ca	1.60
21	*ret*	Ret proto-oncogene receptor tyrosine kinase	1.59
22	*foxa*	Forkhead box A sequence	1.58
23	*si:ch211-56a11.2*	Protein phosphatase 1 regulatory subunit 17	1.54
24	*pclob*	Piccolo presynaptic cytomatrix protein b	1.52
25	*ggctb*	Gamma-glutamylcyclotransferase b	1.43

Average log-fold change indicates higher expression over other cell types. The adjusted P-value for each gene was < 0.05.

### Contrasting expression profiles between NEC and neuron clusters

**Figure 4:**
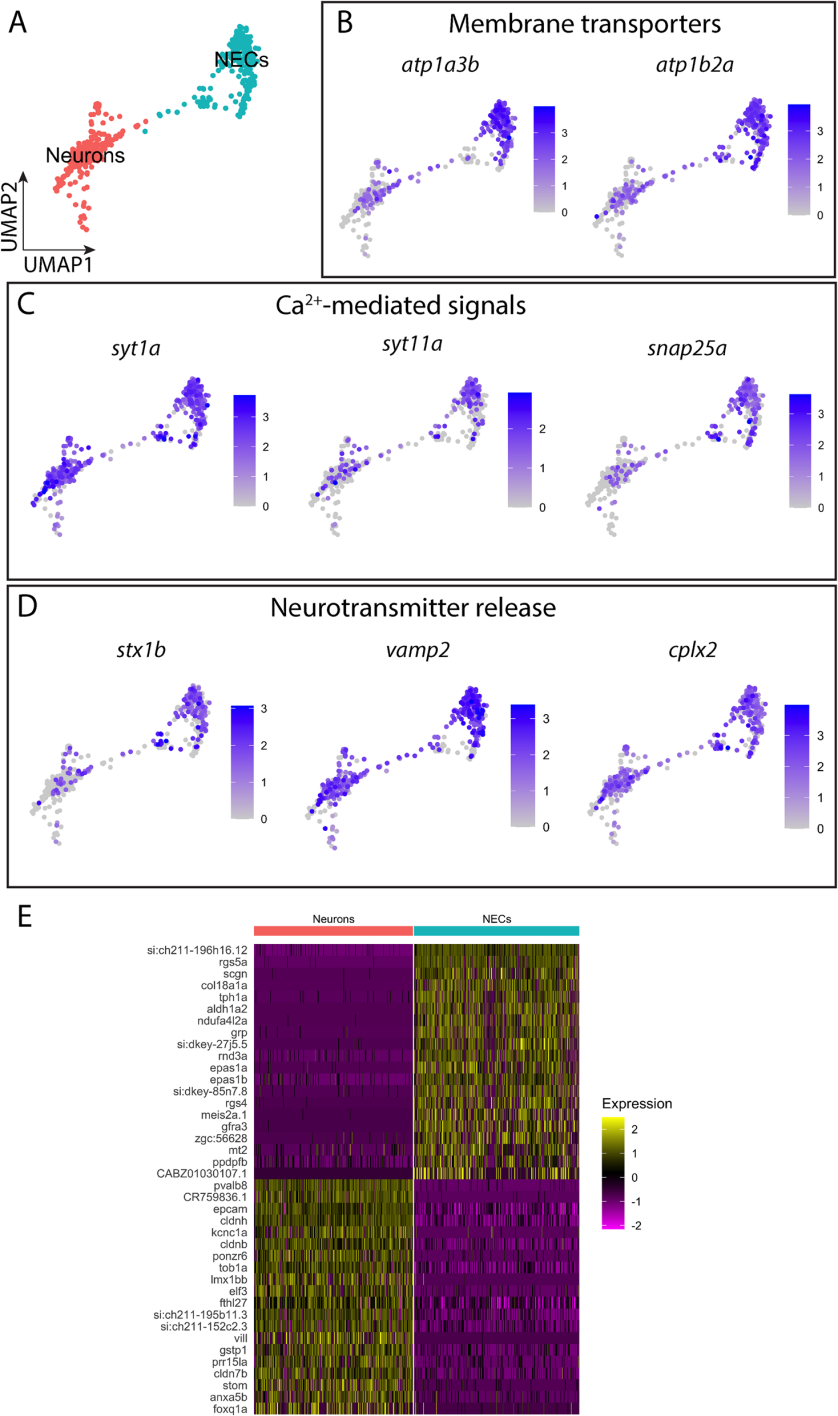
Comparison of gene expression between the NEC and neuron clusters. (**A**) UMAP plot showing the subset of the cell population containing NECs and neurons only. (**B**) UMAP plots showing the log-normalized expression of genes encoding membrane transporters. (**C**) UMAP plots showing the log-normalized expression of genes involved in Ca^2^^+^-mediated signals. (**D**) UMAP plots showing the log-normalized expression of genes involved in neurotransmitter release. (**E**) Heatmap of the top 20 highly differentially expressed genes in NECs and neurons. Scaled gene expression is shown by colours, where yellow indicates relatively high expression and purple indicates relatively low expression. See text and Tables 1 and 2 for gene description.

The NEC and neuron clusters were positioned closely in the UMAP reduction and had overlapping gene expression. To examine this in detail, the two clusters were extracted for comparison (Fig. 4A). Close examination of the subset data containing only NECs and neurons showed that they shared gene expression characteristics of excitable or neurosecretory cells. Although more prevalent in NECs, both cell clusters exhibited profound expression of membrane transporters, such as subunits of Na^+^/K^+^-ATPase, *atp1a3b, atp1b2a* and *atp1a3a* (Fig. 4B). Genes encoding the calcium binding protein synaptotagmins*, syt1a* and *syt11a*, were expressed highly in NECs and neurons (Fig. 4C). In addition, synaptosomal-associated protein (*snap25a*) for exocytotic neurotransmitter release was exclusively found in the two clusters, while the expression level was significantly higher in NECs. Furthermore, a number of genes involved in synaptic vesicle docking or fusion preceding neurotransmitter release, including *stx1b, vamp2* and *cplx2*, were also highly expressed in both clusters (Fig. 4D).

We compared the transcriptomes of both clusters to further investigate unique expression in NECs. DE analysis between the two clusters revealed genes that were differentially expressed in either cluster, and the top 20 genes are presented in a heatmap (Fig. 4E). The results showed that, when compared to neurons, the top differentially expressed genes found in NECs were consistent with previous DE analysis against all other cells. These genes include *rgs5a*, *rgs5al*, *tph1a, ndufa4l2a,* and *grp*. It was also revealed that *epas1a* and *epas1b* were significantly highly expressed in NECs compared to neurons. Moreover, the gene encoding the Ca^2+^-binding protein, secretagogin (*scgn*), was highly differentially expressed in NECs. On the other hand, neurons had high expression of epithelial cell adhesion molecule (*epcam*), claudins (*cldnh, cldnb* and *cldn7b*)*,* delayed rectifier K^+^ channel subunit, *kcnc1a,* and *plac8 onzin related protein 6* (*ponzr6*). When compared to neurons, these genes were down-regulated in NECs.

### Expression of receptors involved in neurotransmission or modulation

**Figure 5:**
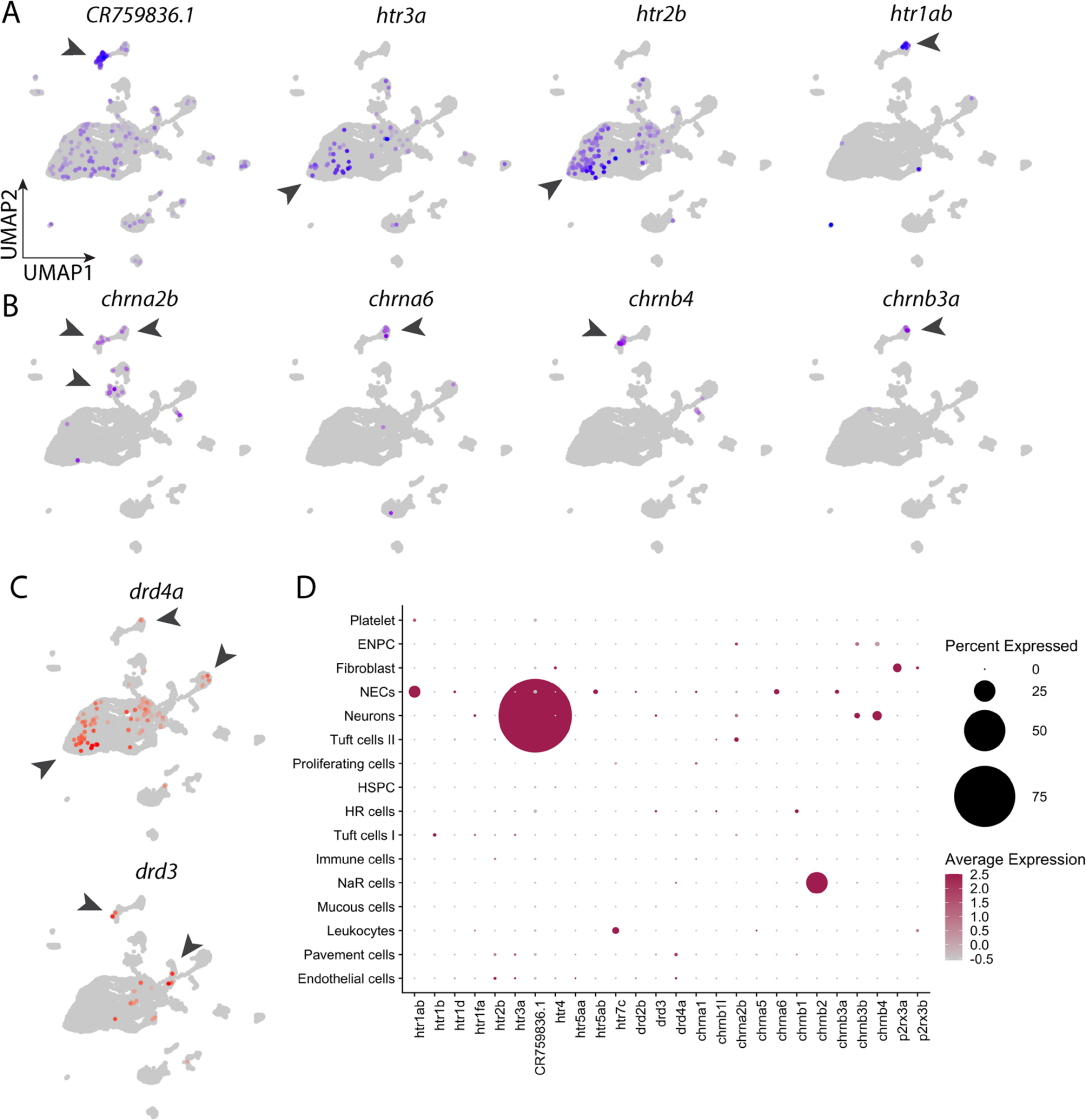
Expression of neurotransmitter receptors in the gill cell atlas. (**A**) UMAP plots showing the distribution of selective 5-HT receptor subunits in all cell populations. Arrowheads indicate cell clusters with significant gene expression. (**B**) UMAP plots showing distribution of selective cholinergic receptor subunits in all cell populations. (**C**) UMAP plots showing distribution of selective DA receptors in all cell populations. (**D**) Dotplot of 5-HT, cholinergic, dopaminergic and purinergic neurotransmitter receptor subunit expression in 16 gill cell clusters. Colour intensity is proportional to the level of expression, and the size of dot indicates the percent of cells showing gene expression in the cell cluster. All dots were manually enlarged to make receptors of lower expression more visible. Scales are provided for percent and average expression. See text and Tables 1 and 2 for gene description.

The present study also mapped the distribution of genes encoding neurotransmitter receptors in gills. Only receptors showing significant expression were selected to be shown in Fig. 5A–C. 5-HT receptors were found throughout many clusters. In particular, *CR759836.1* (encoding 5-HT_3A_-like receptor) was highly expressed in neurons, occupying more than 90% of the cells in that cluster (arrowhead, Fig. 5A). The average expression of *CR759836.1* in neurons was also significantly higher than in other gill cells, with a difference of more than a 4 log-fold change (Table 2). On the other hand, *htr3a* was found in some endothelial cells, tuft cells and pavement cells. Moreover, *htr2b* was expressed in many endothelial cells, pavement cells and tuft cells. The high expression of *htr2b* appeared to concentrate in the endothelial cell cluster (arrow, Fig. 5A). The gene for inhibitory 5-HT_1A_ receptor, *htr1ab*, was found to be most abundant in NECs (arrow, Fig. 5A) and platelets, but nearly absent in other clusters.

In contrast to 5-HT receptors, expression of cholinergic receptors, targets of ACh, was less abundant and generally restricted to NECs and neurons. The nicotinic ACh receptor α2b subunit gene, *chrna2b*, was limited to NECs, neurons and two tuft cell clusters (arrowheads, Fig. 5B). The α6 subunit, *chrna6*, showed expression primarily in NECs (Fig. 5B). The β4 and β3a subunit genes for ACh receptors, *chrnb4* and *chrnb3a*, were present mainly in neurons and NECs, respectively (Fig. 5B).

The presence of all DA receptors was investigated; however, only genes for receptors in the D_2_-like family were detected. The expression of D_4_ receptor, *drd4a*, was found in a few cell clusters, including NECs, endothelial cells and NaR cells (arrowheads, Fig. 5C). In addition, *drd3* was highly expressed only in neurons and HR cells clusters (Fig. 5C).

The dotplot in Fig. 5D summarizes the expression of 5-HT, ACh, DA and purinergic (P_2_X_3_) receptor genes. NECs and neurons were among the top clusters showing the greatest number of different receptors. It could be observed from the graph that *CR759836.1*/*htr3al* was the most abundant receptor gene in neurons in terms of average expression level and percent of cells showing such expression in the cluster. Other neurotransmitter receptors were less abundant but present in cell populations, e.g. expression of *htr1d*, *5htr5ab* and *drd2b* in NECs, and *chrnb3b* in neurons.

### Changes of cell proportion and transcription in chronic hypoxia

**Figure 6:**
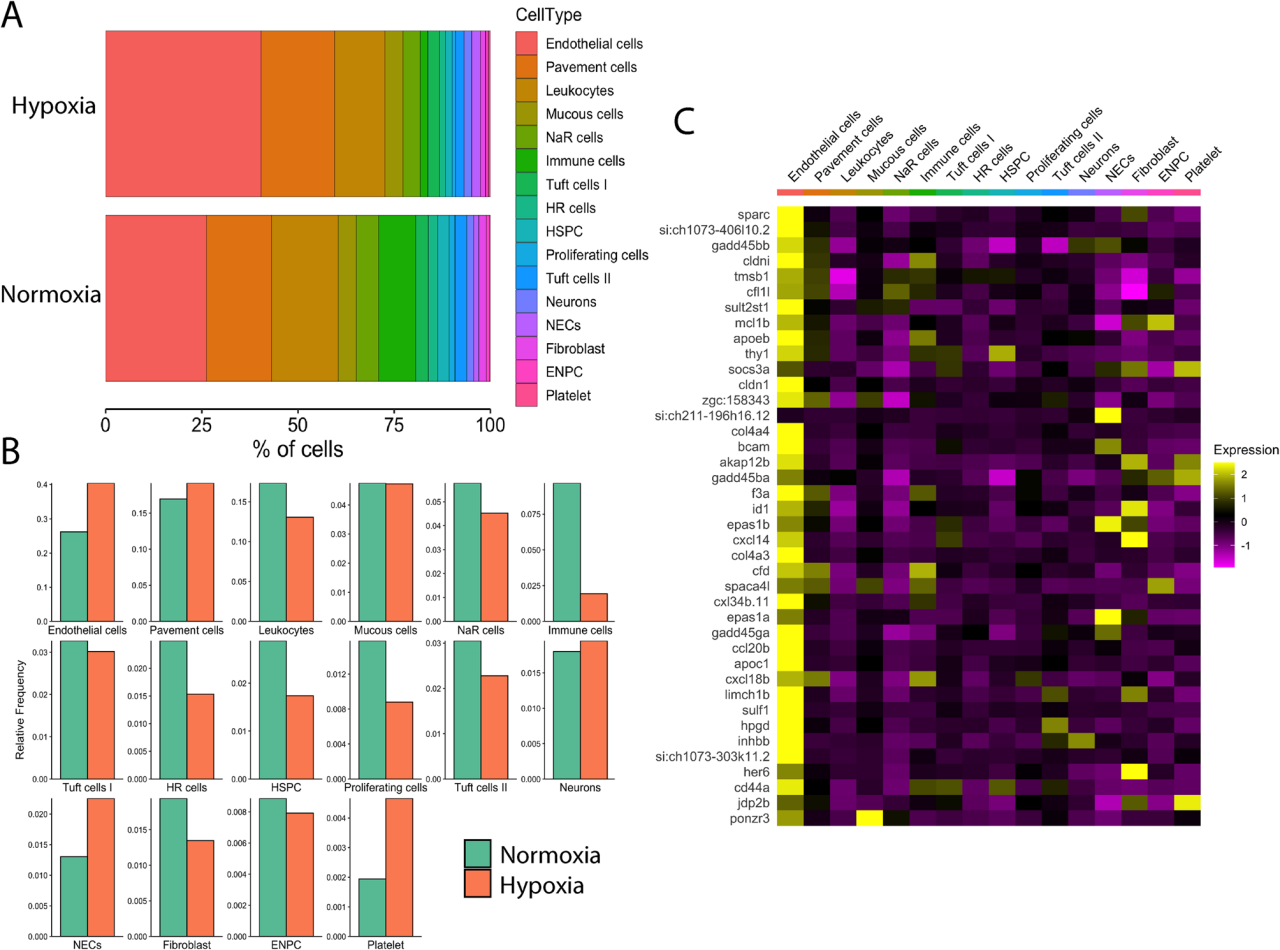
Effects of chronic hypoxia on gill cell composition and global gene expression. (**A**) Stacked bar graph shows the relative difference in % cell composition between normoxic and hypoxic conditions. Cell types are coded by colour and are indicated at the right. (**B**) Bar graphs showing changes in relative cell frequency in normoxia (green) vs. hypoxia (orange) in each cell cluster. (**C**) Heatmap showing the distribution of the top 40 highly differentially expressed genes in chronic hypoxia compared to normoxia. Scaled gene expression is shown by colours, where yellow indicates relatively high expression and purple indicates relatively low expression.

The integrated data contained cells from animals acclimated to hypoxia and normoxia. After demultiplexing the attached MultiSeq barcodes, cells from different treatments could be distinguished. Results showed that the 16 cell identities were present in both treatments. However, cells from fish acclimated to hypoxia had different proportions of cell identities compared to fish in normoxia. As shown in the stacked bar plot in Fig. 6A, endothelial cells and pavement cells were the two largest cell populations in both groups. When compared to normoxia, the hypoxia group had greater proportions of endothelial cells and pavement cells that, when combined, accounted for more than 50% of the overall cell population. However, in normoxia, endothelial cells and pavement cells together accounted for less than 50% of the overall cell population. The proportions of individual cell identities are presented in the bar plots in Fig. 6B. Results indicated that 3 cell identities showed an increase in cell proportion in hypoxia. These cell types were NECs, endothelial cells and platelets. In contrast, leukocytes, immune cells, HR cells, HSPC, proliferating cells, tuft cells II and fibroblasts showed a decrease in relative frequency in hypoxia. In addition, mucous cells, tuft cells I, neurons and ENPC remained largely unchanged in their relative frequency.

To determine the effects of chronic hypoxia at the transcriptional level, we conducted a differential gene expression analysis between hypoxia and normoxia with all cell clusters in the dataset. The top 40 differentially expressed genes in hypoxia compared to normoxia were selected and their expression in each cell identity is shown in the heatmap in Fig. 6C. A full list of all genes that were differentially expressed in hypoxia is provided in Supplementary Table S2. Differentially expressed genes in hypoxia were mostly present in the endothelial cell cluster. Among the top differentially expressed genes in hypoxia across all cell types, many were directly involved in regulation of the cell cycle, such as *gadd45bb, gadd45ba,* and *gadd45ga.* Other differentially expressed genes are expected to contribute to the structure or growth of cells, including *cldni, tmsb1, cfl1l, apoeb,* and *socs3a*. Interestingly, NECs showed the highest expression of several genes that were upregulated by hypoxia, including the HIF genes, *epas1a* and *epas1b*, and the G-protein signaling regulator, *si:ch211-196h16.12*. We did not detect significant hypoxic upregulation of gene expression in neurons.

### Differential gene expression analysis in NECs exposed to chronic hypoxia using DEsingle

**Figure 7:**
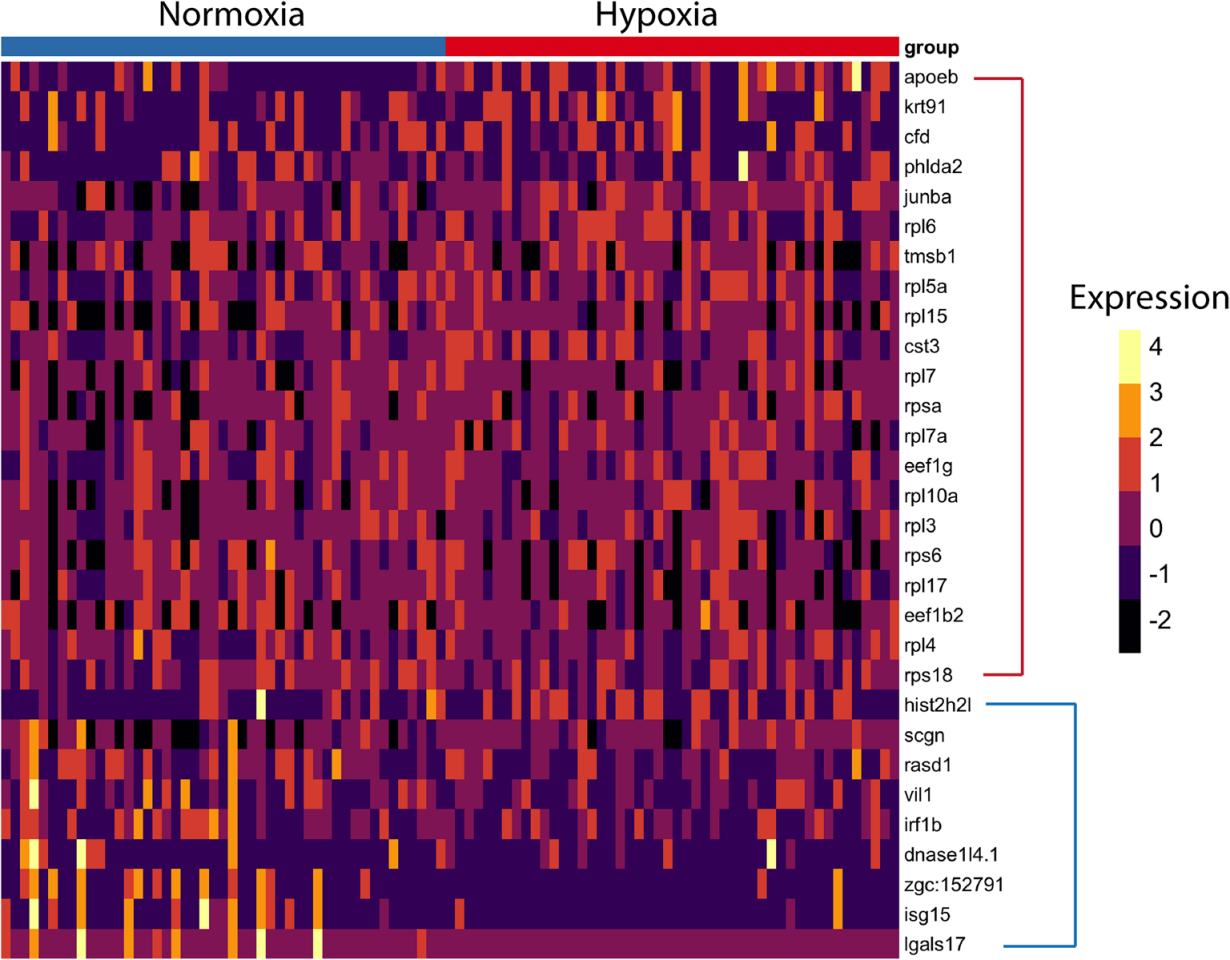
Differential gene expression analysis of NECs between normoxic and hypoxic conditions using the DEsingle method. Heatmap of highly enriched genes that were statistically significant (P < 0.05) in both normoxic and hypoxic conditions in the NEC cluster. Genes enclosed in the red bracket were significantly expressed in hypoxia. Genes enclosed in the blue bracket were significantly expressed in normoxia.

To further examine the effects of chronic hypoxia on transcription in NECs with a higher level of accuracy, differential gene expression analysis was conducted using DEsingle with the subset data containing only NECs. The results revealed that 30 genes were significantly differentially expressed in cells from hypoxia compared to normoxia (adjusted P-value < 0.05). Among these genes, 21 were upregulated (within red bracket, Fig. 7) and 9 were down regulated (within blue bracket, Fig. 7) in hypoxia. The top highly regulated genes in hypoxia included *apoeb, krt91, cfd, phlda2, junba*; ribosomal proteins, such as *rp7, rpl10a, rpl6, rps6*; and elongation factors, *eef1g* and *eef1b2.* In particular, *phlda2* is believed to be involved in the positive regulation of apoptosis^45^, whereas *junba* is a proto oncogene, which could contribute to cell proliferation^46^. Most of the genes that were highly upregulated were encoding ribosomal proteins or elongation factors. On the other hand, genes that were down regulated in hypoxia included *lgals17, isg15, zgc:152791, dnase1l4.1, irf1b, vil1, rasd1, scgn* and *hist2h2l*. Aside from *rasd1* and *scgn,* no other genes involved in the chemosensory transduction of O_2_ signalling were found to be differentially regulated after 2 weeks of chronic hypoxia.

## Discussion

Investigating the mechanisms of O_2_ sensing and chemotransduction in the fish gill has been limited due to our lack of understanding of the cell types involved and the molecular basis of these processes. Using a transgenic zebrafish, the present study employed single-cell RNA sequencing and developed a transcriptomic atlas of cells from the gills. The single cell atlas presents information regarding gene expression profile, cell identification, and transcriptomic similarities among 16 different cell populations, including chemoreceptive NECs, neurons, endothelial cells and ionocytes, using unbiased clustering analysis. We were able to identify expression of numerous genes implicated in O_2_ sensing and chemosensory signaling, including those involved in neurotransmission, neurosecretion, and those encoding ion channels and mitochondrial proteins. Moreover, we demonstrated transcriptional changes in gill cells following exposure to chronic hypoxia.

### Transcriptomic profiling of chemoreceptive neuroepithelial cells

In the cell atlas, chemoreceptive NECs were transcriptionally similar to the neuron population and separated from other gill cells. NECs of the fish gill are considered to be neuron-like, or paraneurons^47^. NECs showed a unique transcriptome and genes involved in 5-HT production, such as *tph1a*, were among those highly expressed. In zebrafish, three paralogs of *tph* genes are present in the central nervous system: *tph1a*, *tph1b* and *tph2*^48^. In a recent report, *tph1a* expression was described in cutaneous NECs and cells of the pharyngeal arch in zebrafish^49^. The present results of *tph1a* expression in O_2_-sensitive NECs of the gill filaments provide evidence that 5-HT is produced within NECs, and support accumulating evidence that 5-HT is an important neurotransmitter involved in O_2_ sensing in the gill^11,17–19^. In addition, the high expression of *slc6a4a*, which encodes the serotonin transporter (SERT) and displays broad expression in the zebrafish brain^50^, may indicate reuptake of 5-HT by NECs from the synaptic cleft after the release of 5-HT during signal transmission. Furthermore, the gill cell atlas recapitulated expression of *slc18a2* in the NEC cluster, confirming the identity of these cells. The *slc18a2* gene has previously been characterized in gill NECs in zebrafish^16^ and encodes a vesicular monoamine transporter involved in storage of 5-HT.

In a recent study, in which single-nuclei RNA sequencing was used to obtain a transcriptomic profile of gill cells in Atlantic salmon^51^, a cell cluster was assigned the identity of “neuroepithelial cells”. In that study, however, the authors did not identify markers associated with chemosensory NECs of the gill epithelium, but instead characterized the cell cluster based on expression of genes associated with cells derived from the neuroepithelium that are uniquely involved in neural development. Such cells are also called neuroepithelial cells but, unlike gill NECs, are involved in the production of migratory cells of the neural crest^52^. While there is evidence of neural crest cells in the gills of zebrafish^53^, chemosensory gill NECs appear not to be associated with the neural crest^13^. It is presently unclear whether the cell cluster described in West et al.^51^ would fit with our model of chemosensory NECs.

In the present article, NECs showed high expression of mitochondrial genes, *ndufa4l2a* and *si:dkey-85n7.8* or *cox8al* (cox8a-like subunit). The mitochondrial electron transport chain (ETC) subunits, NDUFA4l2, COX4I2 and COX8B, were highly expressing genes found in mammalian chemoreceptive type I cells^22,24^. COX4I2 and COX8B subunits were suggested to interact to regulate O_2_ diffusion across the mitochondrial inner membrane, a process that can be highly sensitive to a decrease in PO_2_, marking a potential site of acute O_2_ sensing in these cells^22^. The highly expressed cox8a-like subunit in NECs in zebrafish could pose a potential site of O_2_ sensing. In mouse, the COX8B gene contained HIF binding sites in the promoter region and was proposed to be overexpressed due to high expression of HIF2α genes^22^. In addition, expression of *Epas1*, which encodes HIF2α, was necessary for acute O_2_ sensing by carotid body type I cells as well as the ventilatory sensitivity to hypoxia in mice^54,55^. In our dataset, we observed concurrent high expression of the Hif2α genes, *epas1a* and *epas1b* in zebrafish NECs, suggesting a key role of HIF2α in acute O_2_ sensing during vertebrate evolution.

Differential expression analyses showed that NECs had high expression of genes involved in membrane potential regulation, Ca^2+^ signaling and neurosecretion, compared to other cell clusters. These observations are aligned with the putative O_2_-sensory and neurosecretory roles of NECs^3^. Many highly differentially expressed genes in NECs overlapped with those found in carotid body O_2_-chemoreceptive type I cells^24^, and support the functional homology between these chemoreceptors. Mammalian type I cells expressed high levels of G-proteins (such as *Gnas* and *Gnb2l1*) and a G-protein regulator (*Rgs4*). Similarly, in NECs, pronounced expression of G-proteins (*gng13b, rasd1*) and G-protein regulators (*rgs5a*, *rgs4*) were observed. G-protein subunits and coupled receptors have been found in NECs of the obligate air-breathing fish, *Arapaima gigas*^56^, though G-protein-mediated signaling in fish species remains poorly studied. In the mammalian carotid body, G-protein-coupled receptors, including those activated by 5-HT, were observed to modulate type I cell membrane potential and hypoxic sensory transduction or transmission^57,58^. Our observations of high expression of G-protein regulators could suggest sensory modulation by G-proteins in NECs. In addition, NECs had an exclusively high expression of retinaldehyde dehydrogenase (*aldh1a2*). Retinaldehyde dehydrogenase catalyzes the chemical reaction to produce retinoic acid, which is important for the growth and development of many cells^59^. The retinoic acid receptor gene, *rarga*, was found in all clusters in our dataset. The specific role of retinaldehyde dehydrogenase in NECs is unclear but its high expression could indicate a potential retinoic acid signaling pathway through which NECs communicate with other cells.

### Receptor expression in neuroepithelial cells and neurons represents potential targets for neurotransmission or modulation

Our analysis revealed a distinct neuron population, whose transcriptome was similar to NECs. Their high expression of membrane Na^+^/K^+^ pumps, Ca^2+^ binding proteins and synaptic proteins are indicators of critical processes involved in neuronal function, such as regulation of transmembrane ionic gradients, and Ca^2+^-dependent neurotransmission. This cluster of cells may include serotonergic neurons that have been described previously in the zebrafish gill filaments^12,16^, as some cells within the neuron cluster showed expression of 5-HT synthesizing enzyme genes, *tph1a* and *ddc.* However, the specific function and role of the whole neuron cluster would require further investigation.

Despite their ambiguous identity, these neurons are candidates for involvement in post-synaptic, hypoxic signal transmission or regulation, as they express a variety of neurotransmitter receptors. The most highly differentially expressed gene in neurons was *CR759836.1*, encoding 5-HT_3_-like receptors. Because at least one neuronal population innervates NECs in the zebrafish gill^12^, it is plausible that these 5-HT receptors serve as a target of 5-HT secretion from NECs. The 5-HT_3_ receptor is a ligand-gated ion channel mediating fast synaptic excitation^60^. Previous studies showed that application of the 5-HT_3_ receptor agonist, 2-methyl-5-HT, or the antagonist, tropanyl 3,5-dichlorobenzoate, accordingly increased or decreased ventilation frequency in zebrafish^19^. An excitatory signal from NECs to neurons could therefore be mediated via 5-HT_3_ receptors. In addition, a few other cell populations, such as endothelial cells, were also found to express *CR759836.1, htr3a* and 5-HT_2_ receptors (*htr2b*) but to a lesser degree. Whether chemosensory information, such as hypoxia, could also be transmitted via paracrine signaling from NECs to these nearby cell types through such receptors has yet to be confirmed. The inhibitory 5-HT_1_ receptor (*htr1ab*) was found to be mainly expressed in NECs and could present a pathway for negative feedback regulation as part of their chemoreceptor function.

ACh receptors were identified in the cell atlas. Unlike 5-HT receptors, ACh receptor expression was primarily confined to NECs and neurons. ACh receptor agonists were previously found to induce nerve discharge and changes in ventilation via nicotinic receptors in rainbow trout and zebrafish^17,18,20^, indicating an excitatory role. ACh is a major excitatory neurotransmitter released by carotid body type I cells^61^. In zebrafish, the presence of ACh in a distinct cell population was determined by immunolabelling with antibodies against the vesicular ACh transporter, VAChT^62^. These cholinergic cells were found in close proximity to NECs and nerve fibres, and were suggested to modulate the NEC response to hypoxia through release of ACh. Furthermore, in zebrafish, application of nicotine or the antagonist, hexamethonium, led to an increase or decrease of ventilation frequency, respectively^18,20^. In light of previous studies, our finding of nicotinic receptors in NECs and neurons provides evidence to support a model in which VAChT-positive cells release ACh during hypoxic stimulation, leading to subsequent excitatory post-synaptic effects on ACh receptors of neurons, and paracrine effects on NECs.

Dopamine receptor expression was less prominent, compared to other receptor types, but was present in NECs and neurons. The DA receptor genes, *drd4a* and *drd2b* were present in NECs, whereas *drd3* was present in neurons. All three genes belong to the D_2_-like receptor family that mediates inhibitory neurotransmission. D_2_ receptors are found in the carotid body in type I cells, where they serve as autoreceptors, and on afferent nerve terminals; and in both cases, they are involved in inhibitory neuromodulation^61^. The presence of D_2_-like receptors in NECs and neurons, in the present study, may support an inhibitory role of DA in O_2_ sensing in zebrafish. Dopamine was shown to suppress ventilation when applied exogenously in zebrafish^18^.

### Transcriptional changes following chronic hypoxia

Our results from DE analysis revealed that endothelial cells were most affected by hypoxia. Endothelial cells have long been recognized as part of a vascular O_2_-sensing system^63^. Their high expression of HIF genes, *epas1a* and *epas1b*, in the present study indicates their potential ability to respond to hypoxia. Chronic hypoxia induces proliferation and vascular remodeling^64^, and our results of increased expression of cell cycle regulator genes and cell structural components support this notion. Differential expression analysis using the DEsingle method revealed that chronic hypoxia upregulated expression of genes encoding ribosomal proteins or elongation factors in NECs. An increase in expression of these genes could indicate ongoing cell growth or proliferation, as we observed with the increased proportion of NECs in the hypoxia group. Two weeks of chronic hypoxia was previously shown to increase the proportion of EGFP-labeled NECs, cell size and EGFP expression^16^. Although the increase in EGFP expression, which reflects an increase in *slc18a2,* could not be detected in our data, possibly due to the lack of sequencing depth, our results of increased proliferation agree with previous findings. This increased proliferation of NECs in chronic hypoxia may well be associated with the observed high expression of the Hif2α genes, *epas1a* and *epas1b*. In this regard, it is noteworthy that exposure to chronic hypoxia also causes hyperplasia of mammalian carotid body type I cells and this response is dependent on endogenous HIF2α expression in these cells^65^. The DEsingle analysis did not show transcriptional changes in genes expected to be involved in chemotransduction. These may have occurred before the end of the 2 weeks hypoxic treatment, possibly even during the first few days, as 36 h of hypoxic exposure has previously been shown to cause changes in the HIF pathway in zebrafish^66^.

## Conclusion

The current study has presented the transcriptomic profiles of NECs, neurons and other important cell types of the zebrafish gill using single-cell RNA sequencing under normoxic and hypoxic conditions. We confirmed the identity of NECs in our data by using a transgenic line in which *slc18a2* expression could be identified and matched with expression of other genes that were not previously identified in NECs. In addition, differential gene expression analysis revealed potential pathways of hypoxic signal transduction via mitochondrial signature features and markers of neurotransmission in NECs. Identification of the expression of neurotransmitter receptors found in different cell populations provided evidence for putative synaptic or paracrine signaling mechanisms. The gill cell atlas generated in this article presents a valuable resource for future studies on O_2_ sensing in zebrafish and other aspects of the biology of fish gills.

## Methods

### Animals

Transgenic *ETvmat2:EGFP* zebrafish (*Danio rerio*) used to identify O_2_-sensitive NECs^16^ were bred and raised to 12 months before experiments. Fish were maintained at 28 °C on a 14 h-10 h light–dark cycle in the Laboratory for the Physiology and Genetics of Aquatic Organisms at the University of Ottawa. All procedures for animal care and experimentation were performed according to protocol BL-1760, which was approved by the University of Ottawa Animal Care Committee, and following guidelines provided by the Canadian Council on Animal Care (CCAC). In addition, these procedures followed the guidelines provided by ARRIVE (Animals in Research: Reporting In Vivo Experiments).

### Chronic hypoxia

For chronic hypoxia treatment, adult fish were housed in closed, static 2 l aquaria for 14 days. Fish were fed and given water changes twice per day. A Pegas 4000 MF gas mixer (Columbus Instruments, Columbus, OH, USA) was used to mix compressed air and 100% N_2_ and generate the appropriate partial pressure of O_2_ (PO_2_) to deliver to aquaria through suspended air stones. In the hypoxia group, 4 fish were acclimated to severe hypoxia through a gradual decrease in PO_2_ from 158 to 35 mmHg over the course of 1 week. Fish were then kept at 35 mmHg for an additional week. This PO_2_ level was above the critical O_2_ tension for zebrafish (~ 20 mmHg) and has been shown to induce increases in cell size and number in NECs^16^. As a control, the normoxia group had 4 fish kept in aquaria with continuously aerated water throughout the treatment period.

### Cell dissociation sample preparation

After 14 days of hypoxia treatment, adult fish were stunned by a blow to the head and decapitated for tissue collection. Techniques for tissue extraction and cell dissociation were adapted from previous studies^3,16^ and carried out under sterile conditions. In a laminar flow hood, whole gill baskets were removed from the head and quickly immersed in ice-cold phosphate-buffered solution (PBS) containing (mM): NaCl 137, Na_2_HPO_4_ 15.2, KCl 2.7 and KH_2_PO_4_ 1.5; pH 7.8. Gill baskets were rinsed in wash solution containing 2% penicillin/streptomycin (cat. no. 15140122, Life Technologies Inc., Burlington, ON, Canada) in PBS for 10 min. Individual gill arches were separated to expose single filaments. For each gill arch, the distal region of gill filaments, where NECs are most abundant^12^, was selectively removed and subjected to enzymatic dissociation in 0.25% trypsin/EDTA (cat. no. 25200072, Life Technologies Inc.) for 1 h at room temperature. Filament tissue in trypsin solution was minced with fine forceps and triturated with a 1 ml Pasteur pipette in a 15 ml centrifuge tube to facilitate further dissociation. To stop the trypsin reaction, 10% foetal bovine serum (FBS, cat. no. 10438018, Life Technologies Inc.) was added to the solution containing cells. Cells were passed through a 40 μm Falcon cell strainer to remove tissue debris. After 3 min centrifugation at 1300 rpm the cell pellet was rinsed with fresh PBS. Cells were centrifuged again and dispersed in fresh PBS.

Cell samples of two treatments were individually “barcoded” to allow samples to be combined prior to library preparation and sequencing. The multiplexing procedure was carried out according to the MULTI-seq protocol^67^. Cells were first centrifuged to obtain the cell pellet. 150 μl of 200 nM anchor/200 nM barcode solution was added to cells and cells were resuspended. The lipid-modified DNA oligonucleotide anchor and unique oligonucleotides served as barcodes and were bound to cells during this process. Each sample received a different barcode sequence, which allowed computational demultiplexing later on. After 10 min of incubation at room temperature, 200 nM of co-anchor was supplemented to stabilize anchoring of barcodes at the membrane. Cells were incubated on ice for 5 min, then centrifuged at 1300 rpm for 3 min. The cell pellet was washed twice with PBS containing 1% FBS before resuspension in PBS. Cells were counted manually with a hemocytometer and samples were pooled to reach a final concentration of 500–1000 cells/μl.

### 10× genomics single-cell library preparation and sequencing

Samples of dissociated cells with a viability above 89% were submitted to the StemCore Laboratories at the Ottawa Hospital Research Institute (Ottawa, Canada) for preparation and sequencing. For single-cell cDNA library preparation, barcoded cells from hypoxic and normoxic treatments were pooled. 3 samples of 15,000 cells (i.e. a total of 45,000 cells) were then processed with the Chromium Single Cell 3’ Assay (10× Genomics, San Francisco, CA, USA). cDNA synthesis and library construction were carried out according to the manufacturer’s protocol and the resulting cDNA libraries were amplified using PCR. Samples were sequenced on a NextSeq 500 sequencing system (Illumina, San Diego, USA) with a target read of 18,000 per cell.

### Bioinformatic analysis of single-cell RNA sequencing data

The resulting sequencing data was first analyzed using the Cell Ranger pipeline (10× genomics) to demultiplex raw base cell files, perform alignment, aggregate outputs and generate feature-barcode matrices. The reference genome used in alignment was constructed using the Ensembl release 101 *Danio* genome annotation^68^ and the EGFP sequence. Matrices containing gene features and counts of single cells were further analyzed using the deMULTIplex software package^67^ to demultiplex sample barcodes in order to differentiate samples of different treatment groups.

#### Seurat unsupervised analysis

Bioinformatic analysis of the data was carried out using the Seurat package v3.2.1^69^ within R v4.0.2. Cell quality was assessed through a combination of quality control metrics, including total number of expressed genes, number of unique genes and mitochondrial gene content. Outlier cells were defined if they were found outside of three absolute deviations from the median for any metric and excluded from subsequent analyses. Data were normalized and scaled in Seurat using NormalizeData and log normalized using a scale factor of 10,000. Principal component analysis (PCA) was performed on the scaled data and the first 30 principal components were used in the “FindNeighbors” function to determine the Shared Nearest Neighbour (SNN). Using the “FindClusters” function with a resolution of 0.2, cells showing similar expression were clustered. The multidimensional dataset was visualized in a UMAP graph generated with the first 30 principal components. UMAPs allowed graphical presentation of cell clusters with identity labels and visualization of marker gene expression. To determine unique marker genes of each cluster, Wilcoxon sum tests were performed using the “FindAllMarkers” function. Positive markers from the analysis were used to determine cell identity and new cluster identities were assigned using the “RenameIdents” function. In addition, cells were assigned a score for cell cycle using the “CellCycleScoring” function to determine their mitotic state. Heatmaps and dotplots showing gene expression were generated using built-in functions in Seurat.

#### Doublet removal

An additional doublet removal was performed using the DoubletFinder package^70^, after the quality control was performed in Seurat. DoubletFinder performed PCA and used the principal component distance matrix to find each cell’s proportion of k nearest neighbors to predict doublets. A total of 1,148 predicted doublet cells were removed to reduce aggregated cells in the dataset. The returned output was used in subsequent analyses with Seurat.

#### Differential expression analysis using DEsingle

The Bioconductor package, DEsingle v1.10.0, was used to infer transcriptomic changes in hypoxia treatment in comparison to normoxia. DEsingle uses the zero-inflated negative binomial model to discriminate dropout zeros to detect differentially expressed genes with high accuracy^71^. For each cell cluster examined, an expression matrix containing gene counts and a group vector containing group information were created. These two inputs were passed to the “DEsingle” function to conduct the differential gene expression analysis. Only genes with statistical significance (adjusted P-value below 0.05) between hypoxia and normoxia were selected to be presented in heatmaps.

## Supplementary Information

Supplementary Table S1.

Supplementary Table S2.

## Data Availability

The datasets generated during the current study are available in the Gene Expression Omnibus (GEO) repository, National Center for Biotechnology Information (NCBI) [Accession Identification GSE198044].
